# Primary Lymphoma of Bone Presenting as Spindle Cell Neoplasm of the Vertebral Body: A Case Report and Review of the Literature

**DOI:** 10.1155/2015/518307

**Published:** 2015-04-23

**Authors:** Mahakit Inklab, Richard H. Steingart, Jonathan K. Freeman

**Affiliations:** ^1^Department of Hematology-Oncology, Baystate Medical Center/Tufts University School of Medicine, 376 Birnie Avenue, Springfield, MA 01199, USA; ^2^Department of Pathology, Baystate Medical Center/Tufts University School of Medicine, 759 Chestnut Street, Springfield, MA 01199, USA

## Abstract

Spindle cell variant of lymphoma is a very rare but known disease entity that can mimic a sarcoma. Diagnosis can be even more challenging if the only site of the disease is in the bone. We report a case of primary lymphoma of bone with spindle cell morphology which was successfully treated with a combination of surgery, chemotherapy, and radiotherapy.

## 1. Introduction

Primary lymphoma of bone (PLB) is a rare condition that accounts for less than 2 percent of all lymphomas in adults [1] and 3 to 7 percent of primary bone tumors. Approximately two-thirds of PLB cases are classified as diffuse large B-cell lymphoma (DLBCL), while the rest are comprised of other less common histologic subtypes [2]. Spindle cell variant of DLBCL is an extremely rare morphologic manifestation and has been reported mostly in a few specific organ systems, including the nasal and ocular mucosa, the skin, and soft tissues. Lymphoma cells have been reported to assume a spindle cell configuration as they infiltrate osseous tissues [3, 4]. Due to the rarity of PLB, especially with spindle cell morphology, this disease entity can be misdiagnosed as other more common malignant spindle cell neoplasms, resulting in improper treatment and perhaps poor outcomes. To the best of our knowledge, we are the first to report a case of PLB with spindle cell features which was initially diagnosed as poorly differentiated sarcoma of the vertebral body.

## 2. Case Report

A previously healthy 42-year-old man presented with progressively worsening mid back pain of 4-month duration. An MRI of the thoracic spine revealed diffusely altered T2 signal of the T11 vertebral body which extended into the pedicles, without any evidence of a fracture, cord compression, or discrete mass lesion. This finding was very suspicious for malignant infiltration of the spinal bone. The patient did not experience any muscle weakness, sensory loss, unintentional weight loss, fever, or night sweats. The initial physical examination was unremarkable and did not reveal any palpable lymphadenopathy or hepatosplenomegaly. His routine blood work, including a complete blood count, renal function, and electrolytes, was within the normal ranges.

**Figure 1:**
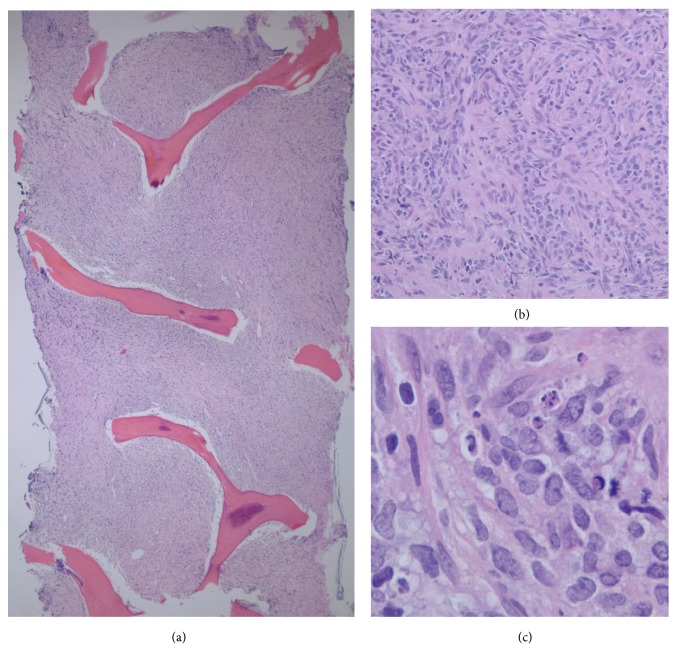
Core biopsy, vertebral body T11. The bone marrow is replaced by a malignant spindle cell neoplasm with streaming and distortion of tumor cells ((a), 4x). Intermediate magnification demonstrates areas of storiform configuration ((b), 20x). High magnification shows spindle cells with apoptosis and mitotic figures ((c), 100x).

The T11 vertebral body lesion was subsequently biopsied under CT-scan guidance. The core needle biopsy specimen showed pathologic findings consistent with high-grade malignant spindle cell neoplasm, favoring poorly differentiated sarcoma with 19 mitoses per 10 high-power fields (Figure 1). The spindle cells were arranged in a nonspecific storiform pattern with necrosis being present in about 50 percent of the tumor cells. Immunohistochemical staining was negative for keratin, S-100, CDK4, and MDM2. On further investigation, 18[F]fluorodeoxyglucose positron emission tomography (PET) CT scan revealed that the only area of hypermetabolic activity was confined to the T11 vertebral body associated with sclerotic deformity. No other areas of mass lesion, adenopathy, or PET activity were appreciated. Given that the pathologic morphology of the tumor was in keeping with sarcoma diagnosis, the patient underwent T11 vertebrectomy and reconstruction surgery via right thoracotomy exposure with curative intent.

**Figure 2:**
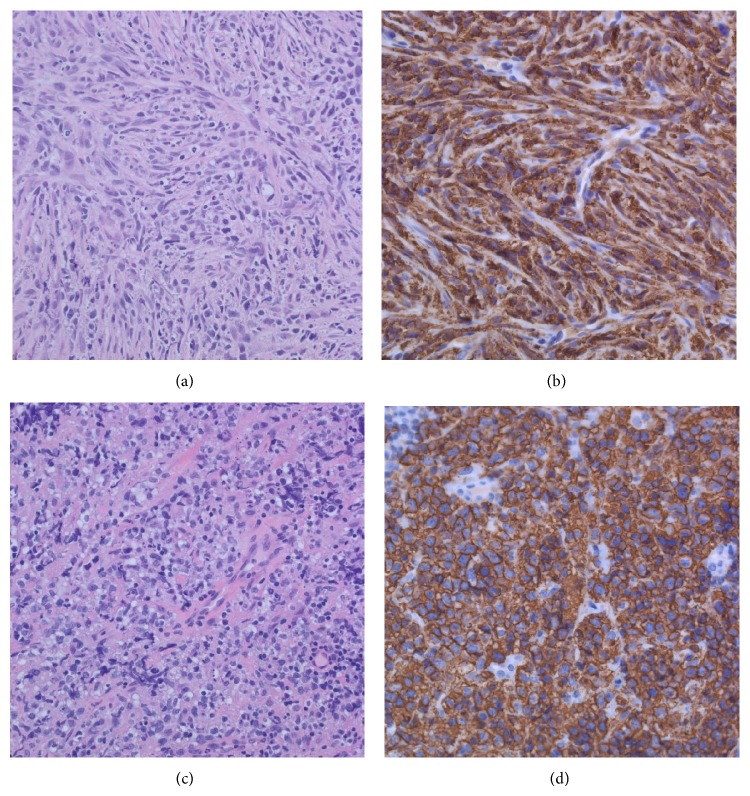
Side-by-side comparison of the biopsy and excisions. Core biopsy ((a), 20x) with CD20 ((b), 20x) compared to the laminectomy ((c), 20x) with CD20 ((d), 20x). The spindle cell's features are pronounced in the core biopsy, whereas the laminectomy specimen is much more typical of lymphoma.

The vertebral body sample obtained from the surgery was submitted for pathological examination. Unexpectedly, the final result revealed DLBCL which consisted of intermediate to large lymphoid cells with open chromatin and variably prominent nucleoli. The tumor was stained positive for CD20, CD79A, PAX5, BCL6, and CD45. Unlike the initial core biopsy sample, this surgical tissue sample did not have obvious spindle cell features. Additional staining which was retrospectively performed on the archival paraffin block from the initial biopsy confirmed that both CD20 and PAX5 were positive in the malignant spindle cells consistent with DLBCL diagnosis (Figure 2). As a part of staging workup, a bone marrow biopsy was obtained from the anterior superior iliac spine and was negative for malignancy.

The final staging was PLB, stage IE, according to Ann Arbor staging system. The patient was treated with three cycles of standard R-CHOP (rituximab, cyclophosphamide, doxorubicin, vincristine, and prednisone) regimen, followed by involved field radiotherapy to 3600 cGy. He tolerated both chemotherapy and radiotherapy well with only transient mild peripheral neuropathy occurring during the treatment. A follow-up PET-CT scan performed one month after finishing treatment was normal without any hypermetabolic activity. The patient continues to do well with no evidence of disease recurrence at the time of this writing which is approximately one year since diagnosis. The total amount of time between the initial diagnosis and the completion of treatment with chemotherapy and radiotherapy was six months.

## 3. Discussion

Lymphomas most commonly originate from lymph nodes, while other primary extranodal sites are much less common. Systemic involvement of the bone can occur in up to 20 percent of the patients with nodal lymphoma [5]. PLB is defined as lymphoma that arises solely from the bone, with or without cortical invasion or soft tissue extension and without involvement of regional lymph nodes or distant visceral organs [6]. This distinct disease entity was first described by Oberling in 1928 [7] and was further characterized as primary reticulum cell sarcoma of the bone by Parker and Jackson in 1939 [8]. With the advancement in immunohistochemical techniques, PLB became better understood and can now be classified into different categories of tumors with lymphoid origin. According to case series by Jawad et al., most cases of PLB are pathologically diagnosed as DLBCL (66.3%) with the rest of the cases falling into the category of other uncommon subtypes of B-cell non-Hodgkin lymphoma [2]. Other variants of PLB, including Hodgkin lymphoma, anaplastic large cell lymphoma, and T-cell lymphomas, are extremely rare and only few cases of each subtype have been reported so far.

The majority of patients present over the age of 30 years, with the median age at diagnosis of 45 years [7]. Men are diagnosed slightly more frequently than women with a male to female ratio of 1.5 : 1 [5]. Most patients present with bone pain which is not relieved by rest. A palpable mass that occurs as a result of soft tissue extension of the osseous disease has been reported in approximately a half of the patients [8]. Less commonly, systemic B symptoms, edema of the affected site, pathologic fracture, and cord compression may also occur at presentation [9]. PLB tends to affect the axial skeleton more frequently than the appendicular sites (62.5% versus 37.5%, resp.) with the majority presenting with single bony lesion (85.5%) as compared to multiple lesions (14.5%) [2]. Patients with PLB should be staged in the same way as any patient with non-Hodgkin lymphoma. Radiotherapy alone was once the standard treatment of PLB which resulted in high level of local disease control (80–100%) but with a high late relapse rate of 50% [10]. CHOP or CHOP-like multiagent chemotherapy with or without radiotherapy is preferred over radiotherapy alone. The addition of rituximab, an anti-CD20 antibody, has also been noted to significantly improve progression-free survival [11]. The 5-year overall survival rate varies depending on the case series and the extent of the disease but is in the range of 76 to 88 percent [12, 13].

The spindle cell variant or sarcomatoid morphology of DLBCL is an extremely unusual finding that can be mistaken for sarcoma or carcinoma [14, 15]. It has been recognized that the lymphoma cells may become spindled as they infiltrate into the bone and soft tissues, suggesting the role of stromal influence as well as cytoskeletal aberrations. Although it has been proposed that the spindling of the lymphoma cells is caused by extensive fibrosis of the surrounding tissue, the exact mechanism of spindling in DLBCL is still undetermined [5, 15]. It has been postulated that lymphoma cells can produce certain cytokines such as tumor necrosis factor (TNF) *α*, platelet-derived growth factor (PDGF), and transforming growth factor (TGF) *β*, among others. These cytokines can induce significant proliferation of fibroblasts which may impinge on lymphoma cells and deform them into a spindle shape [16]. Immunohistochemical staining plays a major role in differentiating spindle variant of DLBCL from sarcoma. CD45, CD20, CD79a, and PAX5 should be positive in DLBCL as in our case, while other markers for sarcoma including smooth muscle actin, muscle specific actin, and desmin should be negative [6]. According to a review of case series by Sugimoto et al. looking specifically at spindle variant of DLBCL, the majority of the cases had disease involvement of the lymph nodes or the skin (23 of 36 cases or 64%) [16]. There were 3 cases that may fit the criteria of PLB with spindle cell features identified in this series. The bones that were involved in these cases were alveolar, maxillary, cranial, and femoral bones. Our case is the first reported case of spindle cell variant of PLB with vertebral bone as the primary and the only site of the disease.

## 4. Conclusion

PLB with spindle cell features is an extremely uncommon disease entity. Due to its rarity, randomized clinical trials addressing treatment options are not available. Treatment with R-CHOP regimen with or without radiotherapy appears to be a reasonable option based on prior experience, resulting in favorable overall outcome. The fact that this particular case initially underwent surgical resection with curative intent made our approach to this rare presentation of lymphoma even more unusual.
